# Behavioral responses of pigs to dietary alternatives to antibiotic growth promoters: a systematic review

**DOI:** 10.1007/s11259-026-11376-0

**Published:** 2026-07-01

**Authors:** Rafaela Nunes dos Santos, Caroline Cintra Rodrigues, Melody Martins Cavalcante Pereira, Leandro Batista Costa

**Affiliations:** 1 Monohub – Research Group for Monogastric Animals, Graduate Program in Animal Science, School of Medicine and Life Sciences, Pontifícia Universidade Católica do Paraná (PUCPR), Curitiba, 80215-901 Paraná Brazil; 2 Graduate Program in Animal Science, PPGCA - School of Medicine and Life Sciences - Pontifícia Universidade Católica do Paraná, Imaculada Conceição Street, 1155 - Prado Velho, Curitiba, Paraná Brazil

**Keywords:** Animal behavior, Feed additive, Pig, Welfare

## Abstract

Intensive swine production stressors induce stress responses that compromise welfare and health, positioning behavior as a critical welfare indicator. Although antibiotics traditionally improve performance, antimicrobial resistance concerns have driven the search for alternatives. This systematic review evaluated the influence of zootechnical additives, used as alternatives to antibiotic growth promoters (AGP), on pig behavior. Following the PICO framework (pigs, antimicrobial alternatives, AGP, behavioral responses), three databases (Scopus, PubMed, Web of Science) were searched. Out of 1,893 identified articles, seven met the inclusion criteria, highlighting the scarcity of standardized behavioral research in this field. Extracted variables included biting frequency, posture (lying/standing/sitting), exploration, agonistic behavior, object interaction, reactivity, stereotypies, and vocalizations. The limited study number and heterogeneous assessment methods restricted direct comparisons between additives. While organic acids showed non-significant effects, probiotics improved exploration and activity in weaned piglets, improved human-animal relationships in sows, and reduced stress reactivity in offspring. L-glutamine increased standing time and novel object exploration in weaned piglets. Regarding phytogenics, *Moringa oleifera* reduced belly nosing, while *Passiflora incarnata* decreased aggression and biting in weaned piglets. In conclusion, feed additives can positively modulate pigs behavioral responses; however, these findings stem from limited data and require cautious interpretation. These findings highlight the potential of specific feed additives to mitigate stress-related behaviors in pigs, while emphasizing the urgent need for standardized ethological protocols to strengthen behavioral evidence across production phases.

## Introduction

Intensive swine production systems, by restricting space and natural behaviors, trigger stress responses that compromise welfare and increase susceptibility to diseases (Hillerer and Gimsa 2024). To mitigate these impacts, antibiotic growth promoters (AGP) are widely used at sub-therapeutic doses, but their indiscriminate use contributes to antimicrobial resistance, threatening therapeutic efficacy in both animal and human health (Rahman et al. 2022).

In 2019, around 4.95 million human deaths were directly related to antimicrobial resistance, making it the third leading cause of death worldwide (Murray et al. 2022). Low- and middle-income countries are the most affected, which raises concern in South America. Despite stricter laws on antibiotic use in some countries, such as the European Union (Ardakani et al. 2023), the search for alternatives is essential to ensure the sustainability of production, animal health, and food safety (Tang et al. 2024).

Among the feed additives currently most used and researched as alternatives to antimicrobials are phytogenics, organic acids, antimicrobial peptides, probiotics, prebiotics, and exogenous enzymes (Ma et al. 2021). These compounds offer multiple benefits: organic acids reduce gastrointestinal pH and modulate the microbiota, improving performance and immunity (Ma et al. 2021); prebiotics and probiotics optimize performance, modulate the immune system, and promote gut health (Liu et al. 2022); phytogenics exhibit antibacterial and antioxidant properties, and enhancing digestibility (Coccimiglio et al. 2016; Su et al. 2019); antimicrobial peptides have antimicrobial activity (Wang et al. 2016); and exogenous enzymes increase nutrient bioavailability, improving digestibility and gut health (Velázquez-De Lucio et al. 2021).

Stressors intrinsic or extrinsic to the animal, compromise general homeostasis (Kraimi et al. 2019). This disruption often leads to the establishment of dysbiosis, resulting from imbalances in microbiota composition and diversity, which in turn compromises intestinal barrier integrity and facilitates the development of pathological states (Kraimi et al. 2019). Furthermore, the bidirectional communication via the microbiota-gut-brain axis (MGBA) suggests that fluctuations in the intestinal microbiota have the potential to alter neuroendocrine signaling. Such changes are reflected in behavioral and physiological parameters associated with welfare, including stress, anxiety, social behavior alterations, and memory (Kraimi et al. 2019). In this context, AGPs mechanism of action may also influence the MGBA by remodeling microbial composition (Muurinen et al. 2021) and metabolic activity (Plata et al. 2022), potentially affecting neurochemical signaling with the pathways that regulate neuroendocrine, immune, and behavioral responses via the microbiota-gut-brain axis (Woś et al. 2025). To mitigate these effects, dietary additives can modulate the MGBA, enhancing nutrient digestibility and immune response while positively influencing the central nervous system and behavioral patterns related to stress and cognition (Kraimi et al. 2019).

Understating animal behavior is fundamental, as it represents the primary indicator of an individual’s internal state (Watters et al. 2021). Behavioral changes such as reduced activity, abnormal aggression or the occurrence of stereotypies may indicate compromised welfare (Deen 2021). Behavioral indicators are highly feasible across farms, zoos, and aquaculture, especially with the increasing integration of automated video and image analysis technologies, as they provide non-invasive and cost-effective assessment tools (Watters et al. 2021). It is important to highlight that animal welfare is intrinsically linked to the sustainable development goals, as improved management practices and effective stress mitigation can enhance productivity while reducing mortality and reliance on antimicrobials. Considering the prevalence of animal-derived products in human diets, these welfare-driven strategies contribute directly to public health and align with increasing consumer demand for antibiotic-free products, offering significant economic advantages and added market value (Mohammadzadeh et al. 2025; Busch et al. 2020).

Numerous studies have investigated the metabolic and immunological effects of feed additives in swine nutrition. However, a clear scientific gap remains regarding their influence on pig behavior, largely due to the lack of standardized methodologies for behavioral assessment. Although antibiotic alternatives have been extensively evaluated for their physiological and health-related outcomes, their behavioral effects, recognized as highly sensitive indicators of animal welfare, are still poorly systematized in the literature. Therefore, considering the importance of behavior as a welfare indicator, the growing demand for antimicrobial alternatives, and the methodological diversity in behavioral evaluations, this systematic review aims to assess the effects of different zootechnical additives used as alternatives to antibiotic growth promoters on pig behavior.

## Materials and methods

Ethical approval was not required for this study, as it consists of a systematic review using exclusively data from previously published research involving pigs.

### Review protocol and research question

A systematic review was conducted on the influence of alternatives to AGP administered via diet on pig behavior. The research question was: “Do dietary alternatives to AGP influence pig behavior?”

### Search strategy

The search, exclusion, and data extraction process was conducted by two members of the research team, and discrepancies were resolved through discussion or the judgment of a third reviewer. A protocol was used for the independent, peer-reviewed literature search, based on the PICO framework. The studied population was pigs, the intervention consisted of alternatives to antimicrobials, the control was AGPs, and the outcome was animal behavior.

The indexing databases used were Scopus, PubMed, and Web of Science. The final literature search was conducted in May 2025. The search string used was as follows: (“swine” OR “pig” OR “pigs” OR “piglet” OR “piglets” OR “gilt” OR “barrow” OR “sow” OR “porcine” OR “boar”) AND (“animal behavior” OR “animal behaviour” OR “behavioral” OR “behavioural”) AND (“alternative to antibiotics” OR “antibiotic alternatives” OR “alternative to antimicrobials” OR “antimicrobial alternatives” OR “growth promoter” OR “growth promoting” OR “additives” OR “additive” OR “feed additive” OR “feed additives” OR “dietary additive” OR “dietary supplement” OR “probiotics” OR “probiotic” OR “prebiotics” OR “prebiotic” OR “essential oil” OR “essential oils” OR “enzyme” OR “enzymes” OR “acidifiers” OR “acidifier” OR “organic acids” OR “homeopathy” OR “plant extract” OR “plant extracts” OR “herbal medicine” OR “phytogenics” OR “phytobiotics” OR “plasma” OR “glutamine” OR “phytotherapeutic products”). A total of 690 articles were found on the Scopus platform, 527 on PubMed, and 676 on Web of Science, totaling 1,893 studies.

### Exclusion criteria by title, abstract, and methodology, and data extraction process

**Fig. 1:**
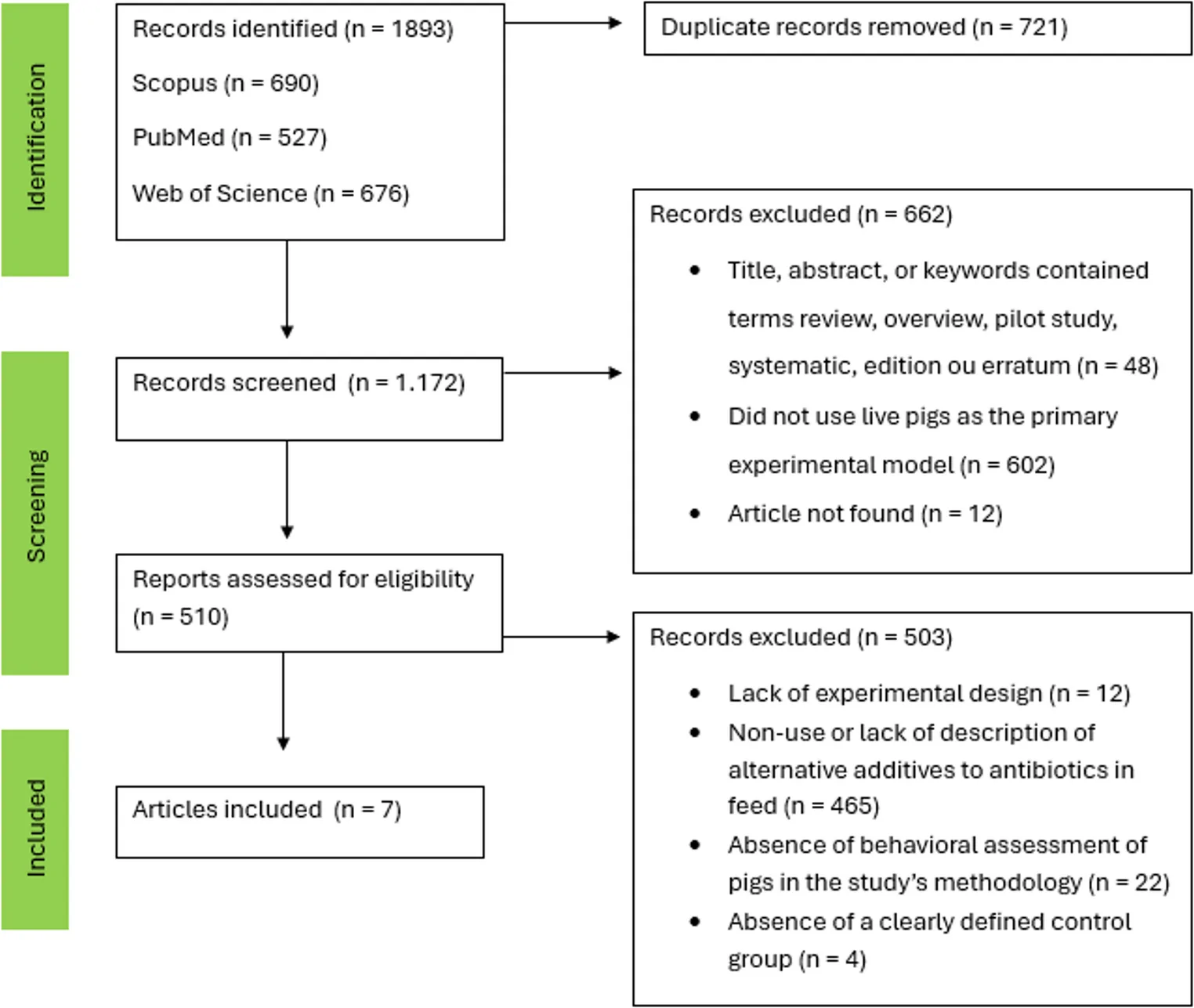
Flowchart of the study search, exclusion, extraction, and selection process

To help with the study selection, all records obtained through the search strategy were exported to the Rayyan platform (Rayyan Management Platform 2024). Initially, 721 duplicates were removed. After duplicate removal, titles and abstracts were screened to identify potentially relevant studies. At this stage, records were excluded if they: (1) were reviews, overviews, pilot studies, chapter books or erratum; (2) did not use live pigs as the primary experimental model; (3) if the article was not found. After applying the criteria, 662 articles were excluded and the remaining articles were then subjected to full-text assessment. During this stage, studies were excluded if they had an absence of an experimental design, did not described an alternative additive to antibiotics in feed, absence of behavioral assessment, or did not include a clearly defined control group. The study selection process is summarized in Fig. 1. With the full-text exclusion step following the previously defined criteria, 503 studies were excluded. This resulted in a total of seven articles included in the systematic review.

The seven articles underwent data extraction, which was performed using a Microsoft Excel for Microsoft 365 spreadsheet, version 2309 (MICROSOFT 2023). The spreadsheet’s structure was based on the following: basic article information (title, authors, journal, year of publication, and DOI), basic study characteristics (study type, sample size, breed/lineage, age, sex and weight of animals, and experiment duration), intervention information (antibiotic alternative used, dose administered, control group, duration of the intervention), observed outcomes (behavior evaluated, description of the behavioral test, frequency and duration of the tests), and study results and limitations.

### Assessment of risk of bias

To assess the risk of bias in the included studies, an adapted table from the RoB 2 tool (Sterne et al. 2020) was used. The reviewers received prior training with practical examples to standardize the assessments. The evaluations were conducted using Microsoft Excel, and the table was generated on the Risk of Bias 4.0 tools platform (Cochrane, London, United Kingdom).

All articles were evaluated based on a judgment (low, some concerns, or high) of individual items within each of the five domains (Table 1).

**Table 1:** Assessment of the risk of bias of the included studies, based on five key domains, and categorization of the risk as Low, Some Concerns, or High for each item assessed

Domains	Key questions	Assessment
1 - Bias due to the randomization process	Did the study describe the randomization method used to allocate animals to experimental groups? / Did the groups have similar characteristics at the start of the study (age, weight, sex, lineage)? / Did the study report how it handled potential imbalances between groups at the start of the study?	**Low**: Yes, the information is clearly described and consistent. **Some concerns**: No complete clarity regarding the randomization or balanced distribution of the animals. **High**: No, there is a lack of information or uncontrolled variation.
2 - Bias due to deviations from the intended intervention	Did the animals receive only the described intervention, without cross-contamination between groups? / Were the diets (control and intervention) administered in a standardized manner in terms of route, frequency, and presentation? / Was there any deviation from the intervention that might have affected the behavioral results? / Did the study clearly describe all the additives used in the intervention, including their doses, composition, and suppliers?	**Low**: No reported deviations, and all parameters were followed according to the protocol. **Some concerns**: Small modifications were identified but reported as having a low impact on the outcome. **High**: Inconsistencies related to the intervention or sampling were detected.
3 - Bias due to missing outcome data	Did the authors describe which animals were included in the behavioral analysis? / Did the study explain and justify any data loss or exclusion of animals? / Did the proportion of missing data differ between groups?	**Low**: All essential data are available. **Some concerns**: Some losses occurred, but they are reported. **High**: More than one critical parameter is missing or was imputed.
4 - Bias in the measurement of the outcome (number of repetitions)	Was the reliability of the observation in collecting behavioral data guaranteed or addressed? / Were the behavioral tests applied under similar conditions for all groups (e.g., time of day, location, duration)? / Did the study clearly describe what behaviors were evaluated, with a methodological definition for each one? / Was the behavioral measurement methodology standardized, validated, or based on reliable previous literature (e.g., ethograms, protocols)? / Did the study specify the timing of the behavioral assessment in relation to the intervention (e.g., beginning, middle, end of the experiment)?	**Low**: Yes, sufficient replicates were reported. **Some concerns**: The number of replicates is slightly below ideal but still allows for some inference, or the description of the replicates is not entirely clear. **High**: No, the number of replicates is insufficient or was not reported.
5 - Bias in the selection of the reported results	Were all the behaviors mentioned in the methodology actually presented in the results? / Is there any indication that the study omitted unfavorable or neutral results? / Were the results presented in accordance with the objectives described in the study?	**Low**: Standardized and recognized analytical methods are used, and the data are appropriately compared side-by-side. **Some concerns**: The description is incomplete, or it is unclear how the behavior was evaluated. **High**: Identification of a potential conflict of interest.

After this assessment, the risk of bias for each article was classified in each of the domains using the following categories:

- Low risk of bias: Studies classified as “low risk” in all evaluated domains.
- Some concerns: Studies that raised “some concerns” in at least one domain.
- High risk of bias: Studies with a “high risk” of bias in at least one domain.

Articles classified as “low risk of bias” were considered to be of high methodological quality, as they presented detailed descriptions of both the methodologies used for behavioral assessment and the interventions applied. Furthermore, they clearly reported the experimental design, explained any deviations from the protocol, and adequately justified the absence of data, providing a comprehensive and transparent presentation of the results.

## Results

A flowchart was designed for better visualization, showing the total number of identified publications and the number of publications filtered at each stage of the systematic review selection process (Fig. 1).

The final database compromised seven studies published between 2017 and 2025, which addressed three phases of swine production: the nursery period, growing/finishing, and reproduction (sows). This indicates that the use of alternatives to antimicrobials in swine production occurs throughout the entire production cycle, making it important to associate the behavior of these animals with the use of these additives. This shows that research in this field is improving due to the substitution of AGP (Tang et al. 2024).

To facilitate the discussion and descriptive analysis of the data obtained in the systematic review, the seven included articles were categorized based on the additives used in the research: organic acids, probiotics, L-glutamine, and phytogenics (Table 2).

**Table 2:** Characterization of seven studies, published between 2017 and 2025, evaluating the behavior of pigs at different phases of swine production, receiving zootechnical additives in their diets

Code	Journal	First Author	Year of publication	Production phase	Number of animals	Additive used	Additive dosage	Control group	Administration period	Evaluated behavior	Additive effect on the behavior
1	Applied Animal Behaviour Science	López-Colom	2023	Nursery	96	Sodium salts of butyric acid and heptanoic acid	3 g/kg	Negative	16 days	Activity behavior, body posture and location in the pen	No effects were observed
2	Translational Animal Science	Barba-Vidal	2017	Nursery	78	Probiotics based on *Bacillus licheniformis* and protected sodium butyrate salt	T1^§^: 1 g/kg of Proporc^#^ T2^§^: 3 g/kg of GustorBP70^¶^	Negative	16 days	Activity behavior and body posture	Effects on activity behavior and body posture
3	Animals	Pereira	2024	Reproduction(Sow)	147	Probiotic^@^	0,05 g/kg	Negative	From the moment of artificial insemination until piglets reach 21 days of age	Sows: Stereotypic behaviors, body posture and human-animal relationship (HAR) observational test Piglets: back test	Sows: Effects on stereotypic behaviors, body posture and human-animal relationship (HAR) observational test Piglets: Effects on the back test
4	Journal of Animal Science	Johnson	2017	Nursery	60	L-glutamine	2 g/kg of L-glutamine	Negative and Positive [chlortetracycline (0,44 g/kg) + tiamulin (0,04 g/kg)]	14 days	Activity behavior and body posture	Effects on activity behavior and body posture
5	Frontiers in Veterinary Science	Parois	2020	Nursery, growing and finishing	246	L-glutamine	2 g/kg of L-glutamine	Negative and positive [chlortetracycline (0,441 g/kg) + tiamulin (0,0386 g/kg)]	14 days	Novel object tests	Nursery: Effect on the interaction with the object Growing: Effect on duration of interaction with the object Finishing: Effect on the latency to interact with the object for the first time
6	Animals	Pastorelli	2022	Growing	120	*Passiflora incarnata* extract	1 g/kg of *Passiflora incarnata*	Negative	28 days	Social and exploratory behavior and Novel object tests	Effects on social and exploratory behavior
7	Tropical Animal Health and Production	Celina	2025	Nursery	16	*Moringa oleifera* leaf meal	50 g/kg of *Moringa oleífera*	Negative	35 days	Activity behavior	Effects on activity behavior

^§^T: Treatments used in the studies; ^#^Proporc: probiotic with 10^2^ cfu/g of Bacillus licheniformis; ¶Gustor2BP70: protected sodium butyrate salt; ^@^Probiotic containing *Lactobacillus acidophilus* (2.06 × 10^8^ CFU/g), *Lactobacillus bulgaricus* (2.06 × 108 CFU/g), *Lactobacillus plantarum* (1.26 × 108 CFU/g), *Lactobacillus rhamnosu*s (2.06 × 10^8^ CFU/g), *Bifidobacterium bifidum* (2.00 × 10^8^ CFU/g), *Enterococcus faecium* (6.46 × 10^8^ CFU/g) and *Streptococcus thermophilus* (4.10 × 10^8^ CFU/g)

Of the seven studies that met all eligibility criteria, the geographical distribution was as follows: Spain: two studies (28.6%); United States: two studies (28.6%); Brazil: one study (14.3%); India: one study (14.3%); Italy: one study (14.3%). This highlights the global relevance of the topic addressed and the search for sustainable alternatives to the use of antibiotics in animal feed.

### Organic acids

Among the selected articles, only one used organic acids as an alternative to AGP. López-Colom et al. (2023) evaluated the behavior of weaned piglets challenged with *Escherichia coli* F4 (1.4 × 10^9^ and 1.5 × 10^9^ CFU for Trial 1 and Trial 2, respectively), receiving sodium butyrate or sodium heptanoate, both protected with a mixture of sodium salts of medium-chain fatty acids distilled from coconut. Behavioral assessment was performed the same way for both trials, using scan sampling to evaluate behaviors related to the disease and resulting from dietary supplementation. The challenged piglets exhibited lethargic behavior, with a higher frequency of time spent lying down. In addition, the animals also visited the feeder area less often and spent more time under the heat source.

In relation to behavioral changes resulting from differences in diet, López-Colom et al. (2023) observed that piglets that received heptanoate spent less time in the feeding area and more time in the lamp area, especially in the afternoon, in addition to being more inactive and more likely to lie down in the afternoon, compared to animals in the negative control and sodium butyrate groups. In the group of animals that received the sodium butyrate-supplemented diet, greater standing activity and more proximity to the feeder were observed, in addition to an improvement in the intestinal barrier through longer intestinal villi and proliferation of caliciform cells.

### Probiotics

Among the studies analyzed, two investigated the use of probiotics as feed additives in pigs. Barba-Vidal et al. (2017) studied the effects of a probiotic based on *Bacillus licheniformis* and partially protected sodium butyrate salt in weaned piglets challenged with *Salmonella Typhimurium*. Behaviors were recorded through scan sampling. Barba-Vidal et al. (2017) showed that piglets fed a probiotic-supplemented diet exhibited more exploratory and active behaviors in the morning, as well as less resting in contact with other individuals. When challenged, the animals showed less activity in the afternoon, with a decrease in positive contact and exploration.

Pereira et al. (2024) investigated the effects of probiotic supplementation, a combination of *Lactobacillus acidophilus*,* Lactobacillus bulgaricus*,* Lactobacillus plantarum*,* Lactobacillus rhamnosus*,* Bifidobacterium bifidum*,* Enterococcus faecium*,* and Streptococcus thermophilus* in the diets of sows, from the beginning of gestation to the end of lactation. In the sows, stereotypical and postural behaviors and human-animal interaction were evaluated, while in the piglets, behaviors were analyzed using the back test. The results showed that the sows that received supplemented diets had better interaction with the handler, as evidenced by a lower aversion to human presence. These sows also remained standing longer and lying down less time compared to the control group, suggesting a higher level of activity and less apathy. Regarding the offspring, piglets from sows that received the supplemented diet vocalized earlier and for a shorter duration during the back test.

### L-glutamine

Two studies evaluated the effects of including L-glutamine in the diet of piglets. Johnson and Lay (2017) investigated the effects of post-weaning transport under heat stress on piglets fed antibiotics (A), no antibiotics (NA), or diets supplemented with L-glutamine (GLU). Weaned piglets were monitored daily by scan sampling, allowing the identification of behaviors indicative of disease. The animals in the NA group showed increased resting time and reduced standing time in the two days following transport. Initially, on days 1 and 2, both GLU and A groups exhibited similar standing and lying behaviors. However, differences emerged in the medium term. On day 7, pigs in the GLU group lay down significantly more than those in group A.

Parois et al. (2020) investigated the relationship between gut microbiota and behavioral responses related to fear and anxiety, as well as assessing the potential of L-glutamine to mitigate the negative effects of stress. New object tests were conducted, considering interaction latency, exploration time, and the occurrence of withdrawal movements (sudden, lateral, or backward movements that occur while the piglet’s head is oriented toward the object) in piglets in the nursery, growth, and finishing phases. L-glutamine supplementation occurred during the first 14 days, with behavioral assessments performed on days 17, 47, 85, and 111 of the experiment. On D17, control piglets showed a tendency to avoid the object less than those receiving supplementation. On D47, no significant effects were reported. On D85, animals that received L-glutamine supplementation or antibiotics spent more time exploring the object, and on D111, they showed lower latency to first contact.

### Phytogenics

Two studies evaluated the use of phytogenics in piglet diets. Celina et al. (2025) evaluated the effects of *Moringa oleifera* leaf meal (MOLM) supplementation in the diet of weaned piglets. Behavior was evaluated through continuous observations 24 h a day for the first seven days and, on day 14, with hourly evaluations for 5 min, observing the variables eating, drinking, lying down, biting, and “belly nosing”. The authors found a reduction in belly nosing behavior in the group that received MOLM supplementation compared to the negative control group.

Pastorelli et al. (2022) investigated the effects of including *Passiflora incarnata* extract in the diet of weaned piglets. Behavior was continuously monitored using all-occurrence sampling over four consecutive three-day periods (days 7–9, 13–15, 20–22, and 25–27), with observations made from 9 am to 12 pm. Aggressive interactions (fighting or displacement, with or without physical contact) and exploratory interactions (sniffing, biting, and chewing elements of the pen, companions, tails, and ears) were evaluated. In addition, the novel object test was applied on three occasions, at nine-day intervals (days 10, 19, and 28), recording the frequency of immobility (animal standing still with head raised and ears alert), mild interaction (superficial exploration, such as sniffing), and intense interaction (active exploration, such as sniffing, chewing, or pushing).

The results indicated aggressive behaviors in the control group compared to the group that received the diet supplemented with *P. incarnata*, as well as greater interaction with environmental enrichment and a lower incidence of tail and ear biting.

### Assessment of risk of bias

**Fig. 2:**
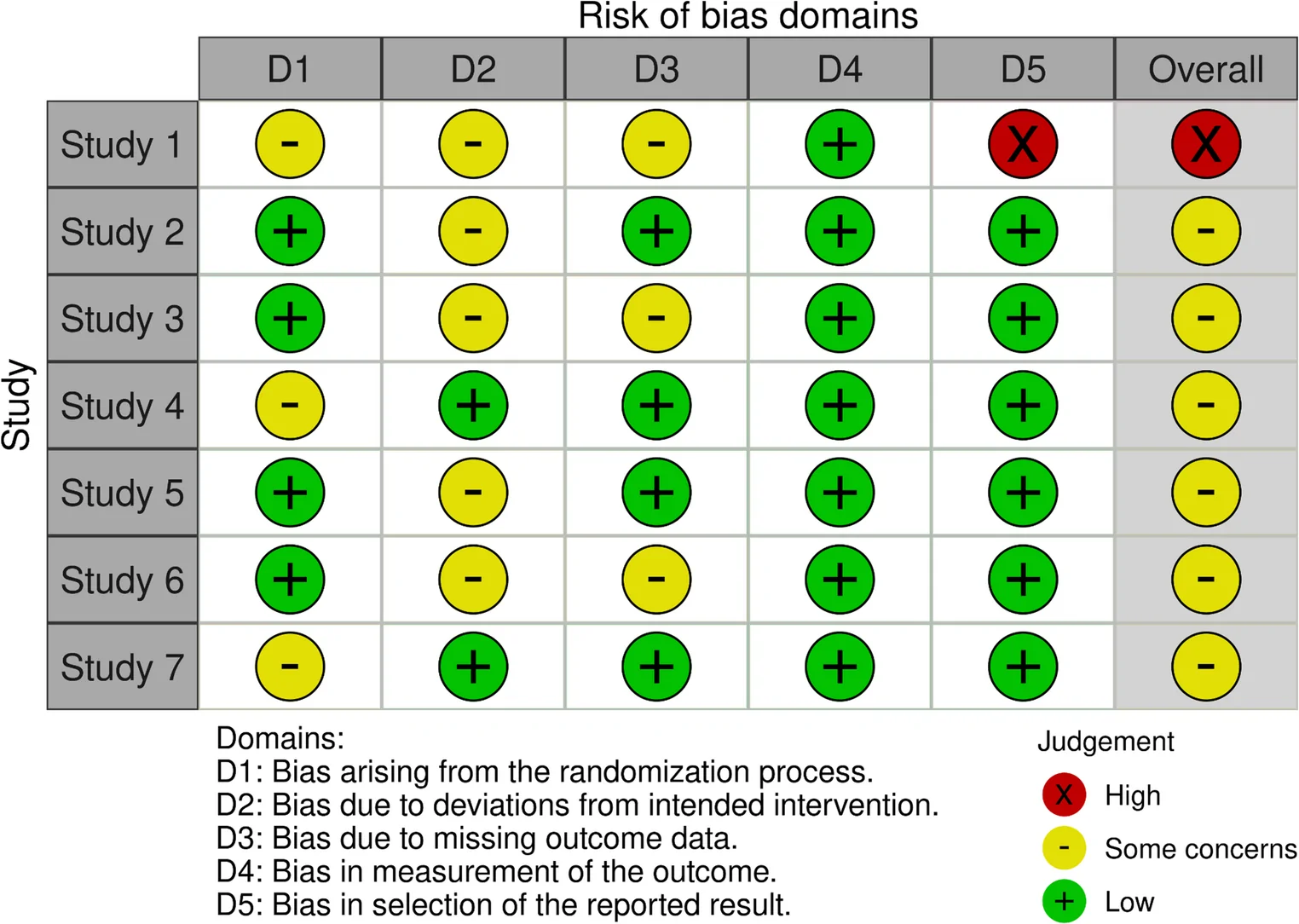
Risk of bias assessment by domain (RoB 2 Tool) in the included studies

The risk of bias assessment was conducted considering five main domains: bias due to the randomization process (D1), deviations from the intended interventions (D2), missing outcome data (D3), measurement of the outcome (D4), and selection of the reported result (D5). The analysis of the seven included studies is reported in Fig. 2.

Among the 7 included studies, all had a low risk of bias in domain D4. Domains D1 and D3 had the highest proportion of studies with a low risk of bias (57.14%). On the other hand, domain D2 had a higher proportion of studies with some concerns, with five of them being classified this way. Regarding domain D5, six out of seven studies had a low risk of bias, and one had a high risk.

Overall, one study was classified as having a high risk of bias, and six studies (85.7%) raised some concerns, such as lack of clear description of the randomization process (animals reported as merely “distributed” among treatments), non-blinded administration of the interventions, and potential deviations from the intended protocol. Additional concerns included unexplained and differential loss of animals between groups and limited transparency in reporting. In some cases, inconsistencies were observed between the narrative description of removals or deaths and the number of animals effectively included in specific behavioral analyses. These results indicate a predominance of methodological limitations among the analyzed studies, particularly regarding deviations from the intended intervention and outcome assessment procedures.

## Limitations

The absence of standardized ethograms and validated behavioral tests limits the comparability and external validity of the findings. A primary limitation of this review is the scarcity of studies for specific production phases, while the majority of the selected studies focused on the nursery phase, there is a significant lack of research regarding the growing and finishing phases and, especially, the reproductive phase (sows), which featured only a single study, which restricts a comprehensive and stage-specific interpretation of behavioral responses. Furthermore, substantial methodological heterogeneity was observed across studies, including differences in observation methods (e.g., scan sampling versus continuous recording), animal age and production stage, sanitary challenge models (studies involving challenged animals versus non-challenge conditions), as well as differences in the duration and timing of supplementation protocols (some studies applying supplementation throughout the entire experimental period and others restricting it to specific phases of the trial.). The use of diverse, non-equivalent behavioral tests and varying additive combinations further complicates direct comparisons. Consequently, a meta-analysis was not feasible due to this heterogeneity and the lack of sufficiently comparable quantitative data. While a risk-of-bias assessment was performed, the inconsistency among studies may have increased the potential for interpretative bias. To mitigate these limitations, comparative analyses were restricted to studies investigating the same additive category whenever possible.

## Discussion

### Organic acids

Organic acid supplementation compensates for low HCl secretion in weaned piglets, reducing gastric pH and activating pepsin for efficient protein hydrolysis. This acidity control optimizes digestibility and establishes a biological barrier against pathogens, preventing unwanted fermentation in the large intestine and the occurrence of post-weaning diarrhea (Suiryanrayna and Ramana 2015). The acidic gastric environment favors beneficial microbiota (e.g., *Lactobacilli*), which inhibits pathogens like *E. coli* through competitive exclusion and lactic acid production. Furthermore, non-ionized organic acids exert direct bactericidal action by penetrating and collapsing the physiology of sensitive microorganisms (e.g., *Clostridia*), preserving microbiota balance and intestinal integrity (Suiryanrayna and Ramana 2015). The inclusion of acidifiers in the diet promotes significant morphological improvements in the intestinal mucosa, with an increase in villus height in the duodenum, jejunum, and ileum (Rathnayake et al. 2021; Wang et al. 2025).

The integrity of the intestinal barrier prevents the translocation of endotoxins (e.g., LPS) that can trigger neuroinflammation and anomalous behaviors, such as states of anxiety and depression. Beneficial microbiota, such as *Lactobacillus*, modulate this axis through the production of Short-Chain Fatty Acids (SCFAs), which cross the blood-brain barrier to exert neuroprotective effects and regulate the synthesis of serotonin—an essential neurotransmitter in controlling aggressiveness. Low concentrations of SCFAs are correlated with behavioral deviations, such as tail biting, highlighting the impact of gut health on the social welfare of swine (Jian et al. 2025).

In the study of López-Colom et al. (2023), the observed higher frequency of time spent lying down is a strategy often observed for maintaining body temperature (Jian et al. 2025). Visiting the feeder area less often and spending more time under the heat source were attributed to water loss caused by diarrhea and the consequent need to maintain homeothermy. These findings can suggest that infected animals exhibit a typical disease behavior pattern characterized by lethargy, increased resting time, and decreased interest in feeding (Nordgreen et al. 2020).

The differences regardless of behavioral changes resulting from differences in diet in the study of López-Colom et al. (2023), the behavioral differences may be related to the interaction between the promotion of intestinal integrity, the inflammatory response, and the microbiota-gut-brain axis (Kraimi et al. 2019). Despite the potential of organic acids to promote antimicrobial action and mitigate inflammatory processes (Yoon et al. 2018), the behavioral effects observed were not significant to confirm their effectiveness as a functional additive in this experimental condition. Thus, the findings indicate that, although promising, organic acids require further studies to validate their beneficial effect on the behavior and welfare of piglets subjected to sanitary challenges.

### Probiotics

The use of probiotics in swine diets plays a fundamental role in modulating gastrointestinal homeostasis, primarily acting on the regulation of the composition and activity of the endogenous microbiota. These beneficial strains, such as *Lactobacillus* and *Bifidobacterium*, promote the competitive exclusion of pathogens (e.g., *E. coli* and *Salmonella*) and strengthen the integrity of the epithelial barrier through the regulation of tight junction proteins (Ding et al. 2021).

The presence of pathogens can lead to negative behavioral changes (e.g., prostration and reduced appetite in cases of *Salmonella*), and probiotics with beneficial actions can reverse these conditions and increase exploratory behavior. In addition to structural support, probiotics and their metabolites, such as butyrate, mitigate oxidative stress and exert an immunomodulatory effect by interacting with the gut-associated lymphoid tissue (GALT). This interaction stimulates the immune response, balancing the production of pro- and anti-inflammatory cytokines and stimulating the proliferation of T and B lymphocytes, resulting in greater immunological resilience and protection of the intestinal mucosa (Ding et al. 2021).

Probiotic supplementation enhances stress resilience and mitigates anomalous behaviors, such as tail biting, by modulating the MGBA. In intensive production systems, the stabilization of the commensal microbiota regulates neuroendocrine pathways, improving swine welfare through the MGBA (Zhao and Ren 2025).

The results by Barba-Vidal et al. (2017) can indicate a beneficial effect of probiotics in reducing stress and promoting welfare, possibly through the microbiota-intestine-brain axis. Such effects are mainly attributed to their mechanisms of action, including the competitive exclusion of pathogen adhesion to the gastrointestinal epithelium, the biosynthesis of potent antimicrobial compounds, participation in the immunomodulation of host defenses, and the reinforcement of intestinal barrier integrity (Anee et al. 2021).

With the challenge, the animals showed a sickness behavior, and the occurrence of diseases in pigs can cause significant changes in the behavior and mental state of the animals, such as an increase in the frequency of lying down behavior (Jian et al. 2025). The results obtained showed an association between the presence of infection and the expression of behaviors that are typical of ill individuals, which are characterized by lower physical activity, reduced positive social interactions, and decreased exploration of the environment. These findings corroborate the results presented by López-Colom et al. (2023).

In this present study, probiotic supplementation promoted improvements in behavioral parameters both before and after the bacterial challenge, indicating its beneficial effect throughout the critical weaning period and during infection. These behavioral improvements may be partially explained by the ability of probiotics to modulate the microbiota–gut–brain axis, influencing the intestinal production of neuroactive compounds such as GABA, dopamine, and serotonin, which play a central role in regulating behavior and cognition (Ansari et al. 2023).

The results presented by Pereira et al. (2024) are in line with a previous study, which indicates that sows subjected to less stressful conditions tend to exhibit a higher frequency of standing behavior (Zhang et al. 2022). Furthermore, considering that fear in pigs is directly related to physiological responses to stress (Scott et al. 2009), the lower aversion to human presence observed can be interpreted as an indicator of reduced fear, consequently reflecting an improvement in animal welfare. The results regarding the offspring can indicate lower reactivity and possible greater adaptation to handling, which may reflect a lower propensity for stress, since low vocalization frequency is considered a positive indicator of animal welfare (Jian et al. 2025).

These findings reinforce the hypothesis of transgenerational or long-term beneficial effects of probiotic supplementation on stress resilience. Thus, it is observed that improving maternal well-being can have an indirect positive impact on the behavior and adaptive capacity of piglets, highlighting the importance of a gestational and lactational environment that promotes well-being, with implications for the management and health of neonates, especially in the face of challenges such as weaning and subsequent infections (Pereira et al. 2024).

The results found in both articles highlight the potential of probiotics to modulate the behavior and welfare in pigs at different production phases, bringing benefits with their use and can be considered as an efficient alternative to AGP.

### L-glutamine

L-glutamine is the most abundant free amino acid in the body and acts as a crucial functional amino acid with various metabolic, structural and regulatory roles (Ji et al. 2019). It acts as the primary oxidative fuel for enterocytes and lymphocytes, being metabolized in the gastrointestinal tract to support renewing cells (Wang et al. 2015). Glutamine is also crucial for regulating cellular redox status by contributing to glutathione production, thereby protecting cells from oxidative stress. Its cytoprotective effects include the inhibition of apoptosis and the induction of heat shock proteins, reducing cellular damage and inflammation (Curi et al. 2005). Dietary glutamine supplementation helps reverse these effects by restoring intestinal morphology, increasing villus height, and reducing crypt depth (Alloui et al. 2013; Hanczakowska and Niwińska 2013). Furthermore, glutamine positively modulates the gut microbiota by promoting beneficial bacteria and reducing pathogenic populations (Alloui et al. 2013). As a result, glutamine supplementation reduces the incidence of post-weaning diarrhea, improves feed efficiency, increases feed intake, and enhances average daily gain, helping piglets overcome weaning stress (Alloui et al. 2013; Cabrera et al. 2013; Ji et al. 2019).

The results from the study of Johnson and Lay (2017) with the animals in the NA group showing increase in resting time and reduction of standing time suggests compromised welfare and manifestation of disease behaviors, as described by López-Colom et al. (2023) and Barba-Vidal et al. (2017). The change in behavior with the pigs in the GLU group laying down significantly more suggests that glutamine may have contributed to a more effective recovery, allowing for a longer period of rest.

In summary, although the initial effects of glutamine and antibiotics were similar, they generated different behavioral responses in piglets in the medium term, highlighting the role of glutamine in modulating recovery and also as a potential of L-glutamine as a functional alternative to the use of AGP. L-glutamine has beneficial effects on the immune system, anti-inflammatory properties (Fan et al. 2023), and contributes to maintaining the integrity of the gastrointestinal tract (EFSA 2009), which may contribute with the results observed.

The results from the study of Parois et al. (2020) indicate that L-glutamine promoted behavioral responses comparable to those of the antibiotic, suggesting its potential as an alternative additive to modulate behavioral responses to stressors, reinforcing its applicability as a functional substitute for conventional antimicrobials. Corroborating these results, one study observed a tendency toward reduced aggressive behavior in piglets that received a diet supplemented with L-glutamine compared to the group that received antibiotics (Duttlinger et al. 2019).

This paper reinforces the role of L-glutamine as an alternative to antibiotics, emphasizing that animal behavior can be modulated in stressful situations, such as weaning, transport, or pathogen challenges. Therefore, it can be concluded that L-glutamine showed similar results to antibiotics in the studies by Johnson and Lay (2017) and Parois et al. (2020), as demonstrated by a positive behavioral result such as increased standing time and decreased lying behavior. These results can indicate a lower expression of behaviors associated with stress, as well as greater interest and interaction with new stimuli, reflecting greater curiosity and exploratory motivation. This ability to explore and engage with the environment is considered a positive indicator of welfare, since healthy animals tend to show interest in their surroundings (Golden and Dilger 2024).

### Phytogenics

Phytogenic additives are natural bioactive compounds that possess antimicrobial, antioxidant, and immunomodulatory properties (Nantapo and Marume 2025). Phytogenics, particularly essential oils, interact with and alter the structure and permeability of bacterial cell membranes, leading to leakage of cellular contents and ultimately microbial death, being especially effective against Gram-positive bacteria (Brenes and Roura 2010; Yang et al. 2015).

At the cellular level, they inhibit the nuclear factor kappa B (NF-κB) signaling pathway, which is responsible for the inflammatory response and the production of pro-inflammatory cytokines such as TNF-α and IL-6, while activating the protective Nrf2 pathway. This increases the expression of endogenous antioxidant enzymes, thereby protecting the intestinal epithelium from oxidative stress (Abdelli et al. 2021; Madesh et al. 2025). In addition to strengthening host immune defenses by increasing the concentrations of key immunoglobulins, such as serum IgG and secretory IgA in the intestinal mucosa, dietary supplementation with phytogenics promotes an increase in villus height and a reduction in intestinal crypt depth. This significantly expands the contact surface area and enhances the nutrient absorption capacity of the intestine (Nantapo and Marume 2025). As a key clinical outcome, a reduction in both the incidence and severity of diarrhea is observed (Madesh et al. 2025).

In the study of Celina et al. (2025), the reduction in belly nosing, a behavior often associated with frustration and stress (Widowski et al. 2008), in the group that received MOLM supplementation, indicates that this supplementation may help relieve post-weaning stress. These results corroborate with literature about the properties of *Moringa oleifera*, which contains bioactive compounds such as flavonoids and polyphenols that have been shown to have anxiolytic properties, which could help reduce stress-related behaviors (Noubissi et al. 2022).

According to the study of Pastorelli et al. (2022), the literature shows that *P. incarnata* has antioxidant and anti-inflammatory properties (Kim et al. 2017) and calming effects (Pastorelli et al. 2020). Thus, the reduction in aggression and abnormal behaviors, such as tail and ear biting, observed in the supplemented group corroborates the potential anxiolytic effect of the extract in weaned piglets.

Both studies used different additives and employed distinct methodological approaches for behavioral assessment, which naturally poses challenges for direct comparison of their results. However, both studies suggest improvements in animal welfare: supplementation with MOLM was associated with a reduction in belly nosing, while *Passiflora incarnata* led to less aggression and greater environmental exploration. These findings reinforce the potential of bioactive compounds present in phytogenics as behavior modulators, with possible anxiolytic effects. However, limitations of these studies include the short duration of interventions and the lack of direct comparisons between different phytogenics, which restricts the generalization of conclusions about their effects.

## Conclusions

The analysis of the seven selected studies reveals that feed additives, such as probiotics, L-glutamine, and phytogenic compounds, can possibly modulate the behavior of pigs subjected to various stressors, including weaning, transport, sanitary challenges, and heat stress, especially during critical phases like the nursery and reproduction. Among the main effects observed are reduced reactivity to maternal stress, greater exploration and activity, improved human-animal interaction, more time spent standing, a reduction in abnormal behaviors (e.g., “belly nosing”), and decreased aggressiveness. However, the methodological heterogeneity among the studies limits a direct comparison of results, highlighting the need for standardized ethological protocols. Also, the scarcity of studies highlights an important research gap in the evaluation of behavioral outcomes of nutritional interventions in pigs. Thus, it is recommended that future investigations prioritize the development and validation of consistent behavioral methods, with clearly defined ethograms and detailed observation and recording protocols. Also, incorporating behavioral outcomes into nutritional research may substantially improve the assessment of animal welfare and guide the development of sustainable feeding strategies in antibiotic-free swine production systems.

## Data Availability

This study is a systematic review based on previously published data that are publicly available in the Scopus, PubMed, and Web of Science databases. No new datasets were generated or analyzed during the preparation of this manuscript. All data supporting this work are appropriately cited within the article.
